# *Rhodolitica* on rhodoliths: a new stoloniferan genus (Anthozoa, Octocorallia, Alcyonacea)

**DOI:** 10.3897/zookeys.1032.63431

**Published:** 2021-04-16

**Authors:** Odalisca Breedy, Leen van Ofwegen, Catherine S. McFadden, Catalina Murillo-Cruz

**Affiliations:** 1 Centro de Investigación en Ciencias del Mar y Limnología; Museo de Zoología, UCR, Universidad de Costa Rica, P. O. Box 11501-2060, San José, Costa Rica; Smithsonian Tropical Research Institute, Republic of Panama; 2 Netherlands Center for Biodiversity Naturalis, P.O. Box 9517, 2300, RA Leiden, The Netherlands; 3 Centro de Investigación en Estructuras Microscópicas, Universidad de Costa Rica, P. O. Box 11501-2060, San José, Costa Rica; 4 Department of Biology, Harvey Mudd College, Claremont, CA 91711-5990, USA; 5 Escuela de Medicina, Departamento de Bioquímica, Universidad de Costa Rica, P. O. Box 11501-2060, San José, Costa Rica

**Keywords:** Biodiversity, Cocos Island, new species, oceanic island, soft corals, taxonomy

## Abstract

*Rhodolitica occulta***gen. nov. et sp. nov.** (Clavulariidae) is described from Cocos Island National Park, Pacific Ocean, Costa Rica. The species was found at various islets and rocky outcrops around the island, 20−55 m in depth. The genus is characterised by tubular, single, erect anthosteles interconnected by thin basal ribbon-like stolons on the surfaces of living rhodoliths. The anthosteles are devoid of fused sclerites, which are only present in the stolons. Coenenchymal sclerites are mostly spindles of various shapes, with a characteristic cylindrical warty type in the outer layer, crosses and radiates. Anthocodiae are armed with points, lacking collarets. Colonies and sclerites are red. Using an integrative taxonomic approach, we separate the new genus from similar genera through both morphological comparison and a molecular phylogenetic analysis. This research is a contribution to the knowledge of the octocoral biodiversity in Cocos Island and marine biodiversity in the eastern tropical Pacific.

## Introduction

Cocos Island, Costa Rica, an oceanic eastern tropical Pacific (ETP) island, has been considered a biodiversity and endemism hot-spot for marine organisms (Cortés 2016). The shallow-water octocoral fauna is poorly represented here, with only three gorgonian species reported, one of them endemic (Breedy and Cortés 2008, 2011). The mesophotic and deep regions of the island have shown a richer unknown octocoral diversity. In 2012, a new family, a new genus, and a new species of Alcyoniina, order Alcyonacea, were described (Breedy et al. 2012) representing the first record of this octocoral group. Every new site or habitat explored around Cocos Island is a potential treasure trove of new species.

In 2007, during a CIMAR expedition aboard the R/V *Proteus*, while dredging at depths of 40–45 m in Chatham Bay, scientists collected rhodoliths with a red stoloniferous octocoral. Rhodoliths are found at several points around Cocos Island (Fernández 2008; Sibaja-Cordero 2012) except in the southwest area. The rhodolith beds extend to 90 m depth, forming dense beds between 20 and 30 m (Cortés 2016). Rhodoliths are composed of several species of calcareous algae and support rich associated biota that ranges from foraminiferans to small fishes (Solano-Barquero 2011).

The Stolonifera include a group of octocorals that consist of individual tubular polyps that arise separately from ribbon-like stolons and present a series of transitional forms, from solitary separated polyps to united polyps joined at their bases in a common extended coenenchyme (Williams 1989; Fabricius and Alderslade 2001). The octocoral group Stolonifera is represented in Cocos Island by the common shallow-water species *Carijoa riisei* (Duchassaing & Michelotti, 1860) in the family Clavulariidae. The stoloniferous octocoral found on rhodoliths was first reported by Breedy and Cortés (2008), tentatively identified as a species of the genus *Rhodelinda* Bayer, 1981, or as a species related to the genus *Paratelesto* Utinomi, 1958. Herein, we describe it as a new genus within the family Clavulariidae. We use an integrative taxonomic approach, combining morphological and molecular analyses in order to phylogenetically position this monospecific species within Octocorallia.

## Materials and methods

### Study site and collection methods

**Figure 1.**
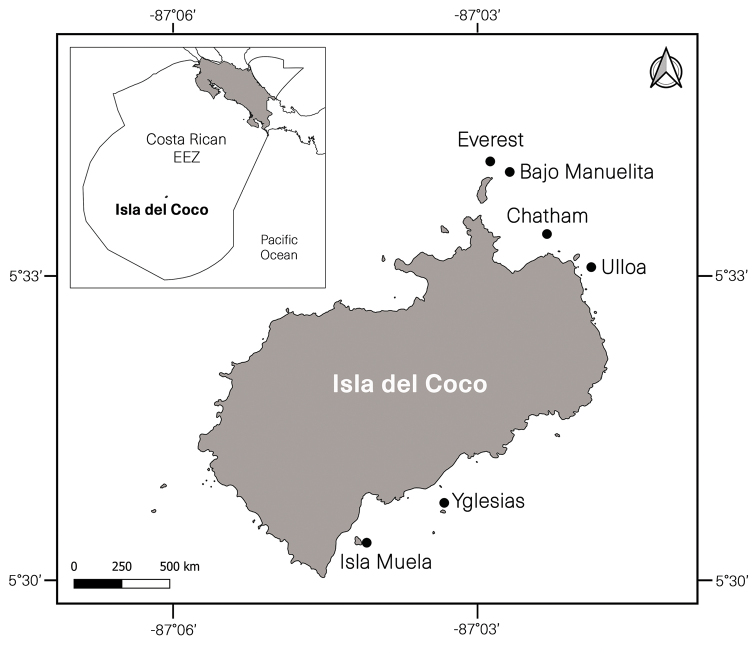
Map showing the collecting sites around Cocos Island, Costa Rica. Map by Beatriz Naranjo, UCR.

The specimens were collected by scuba diving down to 30 m depth, by dredging, and by the submersible HOV ‘DeepSee’ of the M/V ‘Argo’ to 55 m depth. Cocos Island National Park is an oceanic island located between 5°30'–5°34'N and 87°01'–87°06'W in the eastern Tropical Pacific (ETP) approximately 500 km southwest of Costa Rica and more than 600 km northeast of the Galápagos Islands, Ecuador (Cortés 2016). The collected specimens were found on rhodoliths at various points along Chatham Bay (NW of the Island) from Manuelita Islet to Punta Ulloa, and off Manuelita at the Everest pinnacle (Fig. 1). They were preserved in 95% ethanol.

### Morphological analysis

Preserved specimens were morphologically analysed and photographed. For taxonomic identification, sclerites from different parts of the colonies (polyp, coenenchyme, base and stolons) were obtained by dissolving the tissue in 5% sodium hypochlorite; dissociated sclerites were washed several times in distilled water until organic matter was completely removed, dehydrated with 100% ethanol, and subsequently dried in an oven. Sclerites were prepared for light microscopy, mounted in glycerine, and photographed with an Olympus LX 51 inverted microscope. For scanning electron microscopy (SEM), sclerites were mounted on SEM stubs by double stick carbon tape and silver paint, then sputter-coated with gold, 30–60 nm layer, in an Eiko IB-5 Ion Coater; the images were obtained using a Hitachi SEM S-3700N (at 15kV). All specimens were preserved with the anthocodiae partially or totally retracted therefore the description is based on polyp dissection. Measurements of the sclerites were obtained from the SEM images. The holotype and paratypes are deposited at the Zoology Museum, University of Costa Rica, Costa Rica (**MZUCR**).

### Molecular phylogenetic analysis

DNA was extracted from ethanol-preserved tissues with the NucleoSpin® Tissue kit (Macherey-Nagel, Germany) according to the manufacturer’s instructions, and kept at −20 °C until further processing. A partial region of the mitochondrial mismatch repair gene (mtMutS) was amplified with ND42599F (5'-GCCATTATGGTTAACTATTAC-3'; France and Hoover 2002) and MUT3458R (5'-TSGAGCAAAAGCCACTCC-3'; Sánchez et al. 2003), the mitochondrial cytochrome oxidase I gene (COI) with the primers COII8068F (5'-CCATAACAGGACTAGCAGCATC-3'; McFadden et al. 2004) and COIOCTR (5'-ATCATAGCATAGACCATACC-3'; France and Hoover 2002); and the 28S nuclear ribosomal gene with 28S-Far (5'-CACGAGACCGATAGCGAACAAGTA-3') and 28S-Rar (5'-TCATTTCGACCCTAAGACCTC-3') (McFadden and Ofwegen 2012). All the reactions were carried out in 50 μl volume with 10-50 ng DNA, 2.5 units Taq DNA polymerase (DreamTaq, Thermo Scientific, Waltham, MA), 1X DreamTaq Buffer, 0.2 mM of each dNTP, 0.3 μM of each primer and 50 μg of BSA. The amplification protocol for mtMutS consisted of 2 min of initial denaturation at 94 °C followed by 35 cycles of 30 sec at 94 °C, annealing at 50 °C for 30 sec, extension at 72 °C for 30 sec and a final extension at 72 °C for 5 min, and for COI and 28S was 5 min of initial denaturation at 94 °C followed by 35 cycles of 60 sec at 94 °C, annealing at 46 °C for 90 sec, extension at 72 °C for 60 sec and a final extension at 72 °C for 10 min. The resulting PCR products were purified and sequenced by Macrogen Inc. (Seoul, Korea), using the same forward and reverse PCR primers. Sequences from MZUCR2513 have been deposited in GenBank under accession numbers MW491885 (28S rDNA), MW491886 (mtMutS) and MW491887 (COI).

Sequences obtained from MZUCR 2513 were aligned with reference sequences from a wide range of octocoral taxa (Suppl. material 1: Table S1) using the FFT-NS-i method in MAFFT (Katoh et al. 2005). JModeltest (Darriba et al. 2012) was used to identify the best model of evolution for each gene region based on the AIC. GTR+I+G was identified as the best substitution model for mtMutS and 28S, with the similar TrN+I+G suggested for COI. Consequently, GTR+I+G was specified for all three gene regions in both maximum likelihood and Bayesian analyses. Maximum likelihood trees were constructed for each gene region separately using PhyML (Guindon and Gascuel 2003) with 100 bootstrap replicates (Suppl. material 2–4). No conflict was found between tree topologies so all three gene regions were concatenated for subsequent analyses; specimens for which data for one or more genes were missing were not included in the combined analysis. RAxML v8 (Stamatakis 2014) was run with 200 rapid bootstrap replicates, which allows a search for the best-scoring tree and bootstrapping in a single run. Bayesian analyses were conducted using MrBayes v3.2.1 (Ronquist et al. 2012), run for 4 × 10^6^ generations (until standard deviation of split partitions < 0.01) with a burn-in of 25% and default Metropolis coupling parameters.

## Results

### Systematics

#### Class Anthozoa Ehrenberg, 1831

**Subclass Octocorallia Haeckel, 1866**

**Order Alcyonacea Lamouroux, 1812**

**Family Clavulariidae Hickson, 1894**

##### Genus Rhodolitica gen. nov.

Taxon classificationAnimaliaAlcyonaceaClavulariidae

F8747317-1706-5812-A01A-CD0B581E2CD8

http://zoobank.org/12B6B038-9830-4209-94B6-BA453693E194

###### Diagnosis.

Colonies composed of tubular, single, erect anthosteles up to 8 mm tall and closely spaced, 1–3 mm apart forming interconnected groups of 2–15 anthosteles. Anthosteles arise directly from thin basal ribbon-like stolons that encrust irregular surfaces of living rhodoliths. Stolons composed of conspicuous red brittle fused sclerites not extending into anthostelar walls. Bright red coenenchymal sclerites compose the anthostelar walls, including long warty spindles, up to 0.27 mm in length; complexly warted cylindrical spindles up to 0.32 mm long; bent spindles and smaller crosses and radiates. Polyps whitish to transparent. Anthocodiae retractile, armed with eight interseptal points of slender warty spindles, up to 0.24 mm long, and small orange biscuit-like rods along the tentacles. Collaret absent. Collected specimens were preserved with the anthocodiae partially or totally retracted therefore a full description of polyps was not possible.

Colour of colonies bright red. Azooxanthellate.

###### Type species.

*Rhodolitica occulta* spec. nov. by original designation.

###### Etymology.

The generic name is in reference to the substratum to which the colonies were attached: rhodolith rocks.

##### Rhodolitica occulta sp. nov.

Taxon classificationAnimaliaAlcyonaceaClavulariidae

959FBA3C-89C1-5DC1-BE64-56C88EABCF4D

http://zoobank.org/B7B0E8AA-C5DE-433D-A2BC-0E73B386D45E

Figures 2 , 3

###### Material examined.

***Holotype*.**MZUCR 2514, lot 1, ethanol preserved, Cocos Island, Chatham Bay, 05°33.347'N, 87°02.336'W, dredging, 45 m depth, J. Cortés, J. Sibaja-Cordero, Proteus-CIMAR Expedition, R/V Proteus, 13 January 2007. ***Paratypes*.**MZUCR 2514, lots 2, 3, ethanol preserved, same geographical data as the holotype. MZUCR 2734, lot ethanol preserved, Roca Chatham Bay, Langosta, Punta Ulloa, 05°33.222'N, 87°02.053'W, dredging 39–44 m depth, J. Cortés, J. Sibaja-Cordero, Proteus-CIMAR Expedition, R/V Proteus, 17 January 2007. MZUCR 2513 (GenBank accession numbers MW491885 (28S rDNA), MW491886 (mtMutS) and MW491887 (COI)), ethanol preserved, Chatham Bay, Punta Ulloa, 05°33.222'N, 87°02.063'W dredging 45 m depth, J. Sibaja-Cordero, 14 April 2008. MZUCR 3301, ethanol preserved, Bahía Iglesias, Muela Rock, 23 m depth, J. Cortés, 14 January 2007. MZUCR 3302, lot preserved in ethanol, Chatham Bay, 05°32'43.6"N, 87°01'41.7"W, 20 m depth, J. Cortés, 3 April 2009. MZUCR 3303, lot ethanol preserved, off Cocos Island, Everest pinnacle, 55 m depth, HOV DeepSee Dive 2375, A. Klapfer, 25 October 2015.

###### Type locality.

Chatham Bay, Cocos Island, Costa Rica, 45 m depth.

###### Description.

**Figure 2.**
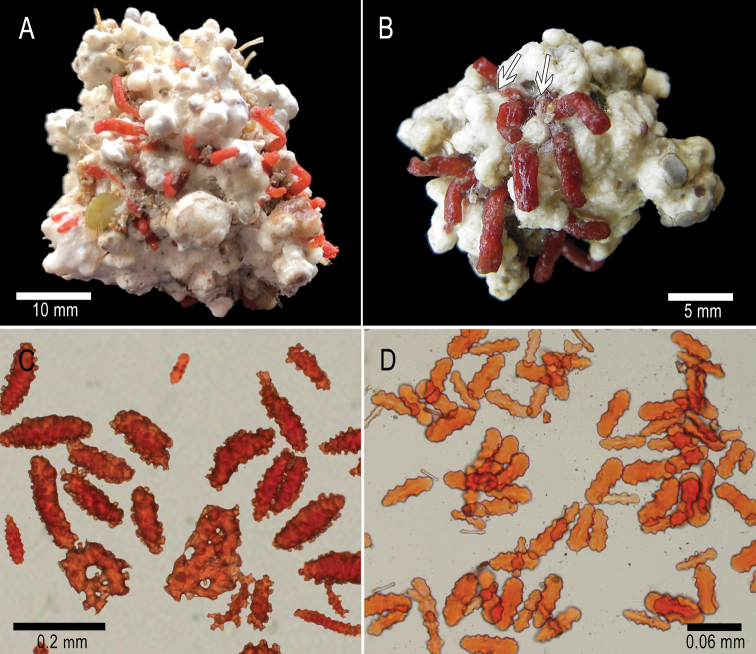
*Rhodolitica occulta* sp. nov. **A** holotype MZUCR 2514 **B** paratype MZUCR 2513, ribbon-like stolons can be observed at the upper cluster of polyps (white arrows) **C** unsorted sclerites of the holotype **D** tentacular sclerites.

**Figure 3.**
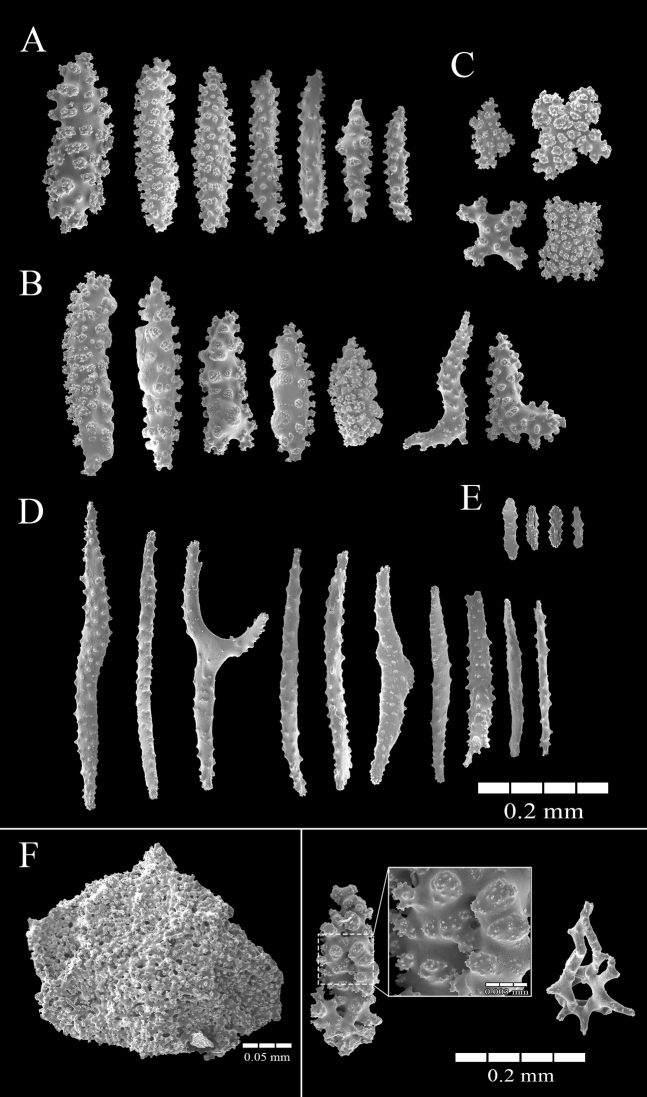
*Rhodolitica occulta* sp. nov., holotype MZUCR 2514 **A, B** outer coenenchymal sclerites **C** inner coenenchymal sclerites **D** anthocodial sclerites **E** tentacular sclerites **F** fused and partially fused sclerites from stolons, details at the right.

The holotype is composed of several bright red tubular, single anthosteles extending up to 8 mm above the surface of a 4 cm-diameter rhodolith (from one lot of five rhodoliths, 4–7 cm diameter) (Fig. 2A). The tubular anthosteles are slightly expanded distally, forming groups of 10–12, and closely spaced, with the bases 1–2 mm apart. The anthosteles arise directly from thin basal stolons (Fig. 2B). The stolons are ribbon-like, approximately 1 mm in width, and extending in irregular patterns on the surface of the rhodolith. Anthostelar walls are composed of bright red, variable coenenchymal sclerites: long warty spindles, 0.17–0.27 mm in length, 0.047–0.07 mm wide (Figs 2C, 3A); complexly warted cylindrical spindles with fused tubercles on one surface, and warty on the other, 0.11–0.32 mm long and 0.045–0.08 mm wide (Fig. 3B); bent spindles, 0.19–0.23 mm long, 0.01–0.013 mm wide (Fig. 3B, end of the row); smaller crosses, radiates up to 0.15 mm long, and transitional forms (Fig. 3C) from the inner coenenchyme. Conspicuous fused or partly fused sclerites are present in the stolons (Figs 2C, 3F); Figure 3F (right side) shows details of sclerites fused together. These brittle sclerites can partially dissociate during the dissolution process, forming small or large aggregations 0.2–1.6 mm long (Figs 2C, 3F). They are present only in the stolons, and do not extend into the anthostelar walls. Anthocodiae are completely retractile into the anthosteles. The anthocodiae have eight interseptal points, composed of slender thorny spindles, 0.23–0.5 mm long and 0.018–0.05 mm wide (Figs 2C, 3D) arranged ‘en chevron’, with very few transverse spindles, not forming a collaret. The points are composed of 10–15 spindles. Orange biscuit-like rods, 0.047–0.095 mm long and 0.014–0.026 mm wide (Figs 2D, 3E) are along the tentacles. Unfortunately, all specimens have been preserved with the anthocodiae partially or totally retracted therefore a full description is not possible.

###### Variability.

The paratypes were found on rhodoliths of 3–9 cm in diameter. Some variation in colour was found in a few paratypes. They present red and orange anthosteles in the same colony. All other characteristics were consistent with those of the holotype.

###### Habitat and distribution.

**Figure 4.**
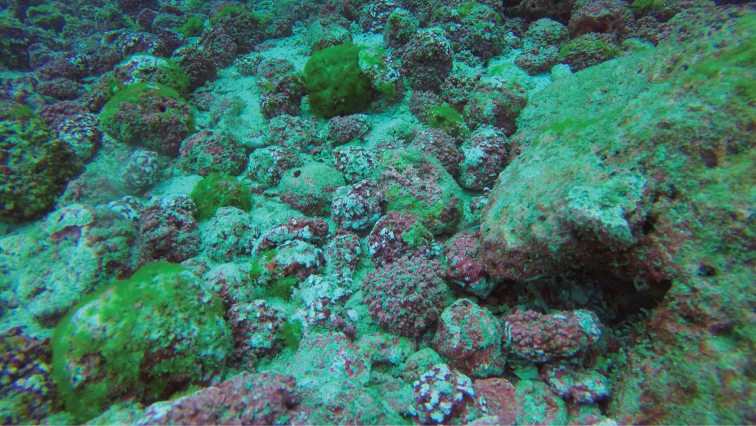
Rhodoliths bed, Pájara Islet, Cocos Island, at 22 m in depth. Photograph by Beatriz Naranjo, UCR.

The colonies were found only associated with living rhodoliths, from 20 to 55 m depth. The rhodoliths were in aggregations, forming extensive beds or dispersed on sandy bottoms (Fig. 4). Specimens were also collected from a dead shell and from a dead piece of coral partially covered by rhodoliths, making it evident that this species is associated with living rhodoliths. In some colonies anthosteles are occupied by several epizoic organisms, especially bryozoans and small hydroids.

The species is presently only known from Cocos Island.

###### Etymology.

An adjective (L), *occultus*, meaning hidden, secret, mysterious. Historically, numerous expeditions to Cocos Island seeking pirate treasures buried somewhere in the mysterious island were completely unsuccessful. The new species, out of sight, hidden amongst rhodoliths, shows that the real treasure of the island is its biodiversity.

### Phylogenetic analysis

**Figure 5.**
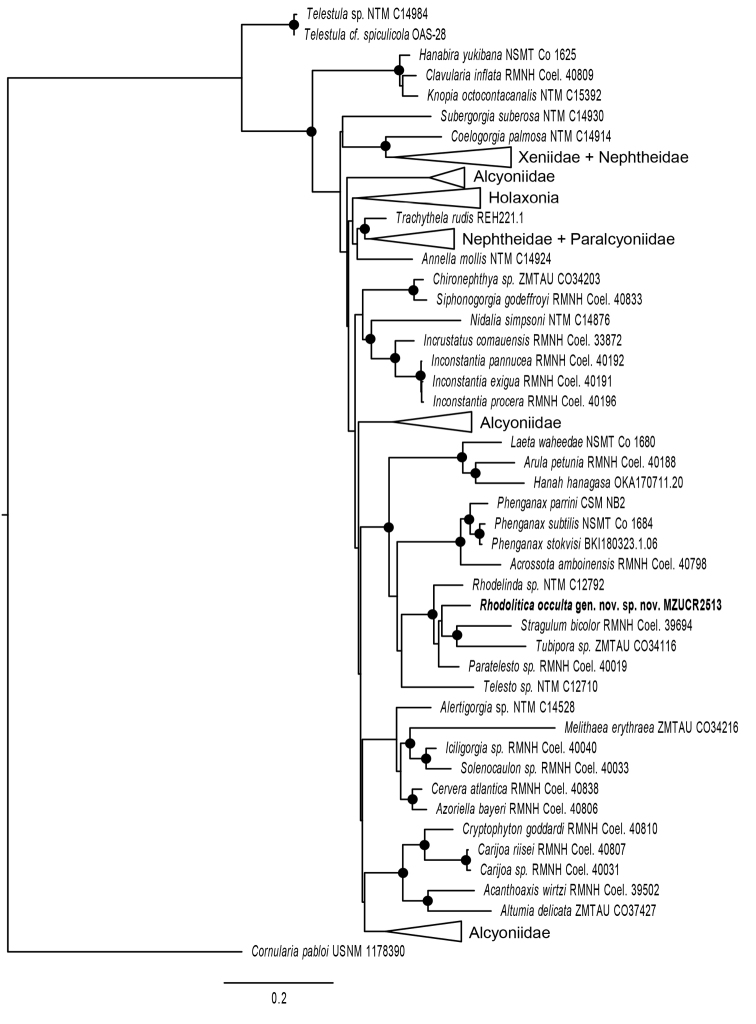
Maximum likelihood reconstruction (2417 nt of concatenated mtMutS, COI, 28S rDNA) of the Holaxonia-Alcyoniina clade of Octocorallia. Two stoloniferous taxa belonging to the Calcaxonia-Pennatulacea clade (*Cornularia*, *Telestula*) were included to root the tree. Clades that did not include any stoloniferans have been collapsed to triangles to facilitate readability. Solid circles on nodes indicate strong support from both maximum likelihood and Bayesian analyses (bootstrap > 70%, pp > 0.95)

All phylogenetic analyses placed MZUCR 2513 in a well-supported clade (ML bootstrap (bs) = 95%; Bayesian posterior probability (pp) = 1.0) that included families Tubiporidae, Acrossotidae, Arulidae, and several genera of Clavulariidae (Fig. 5). Within that larger clade of stoloniferans, both ML and Bayesian methods recovered MZUCR 2513 in a clade with *Paratelesto* sp., *Rhodelinda* sp. and [*Tubipora*+*Stragulum*] (bs = 100%; pp = 1.0) with no support (bs < 50%, pp < 0.90) for the sister relationships among those taxa.

## Discussion

From the morphological point of view, *Rhodolitica* gen. nov. is related to *Rhodelinda*, *Paratelesto*, and *Stragulum* Ofwegen & Haddad, 2011, all within the family Clavulariidae, a conclusion that is supported by the phylogenetic analysis. Morphologically, *Paratelesto* grows in bushy clusters, the colony branches many times, up to four times with secondary polyps (Fabricius and Alderslade 2001); this characteristic separates it from *Rhodolitica* and the other two genera. Although the external layer of large complexly warted cylindrical sclerites in *Paratelesto* is somewhat similar to that in *Rhodolitica* (Fig. 3B), none of the other features are shared. The main difference between the new genus and *Rhodelinda* is that the anthostelar armature in *Rhodelinda* consists of fused sclerites forming a brittle tube (Bayer 1981; Williams 1989; Pastorino and Ituarte 1996). In *Rhodolitica* the fused sclerites are only present in the stolons. In addition, the anthocodial and coenenchymal sclerites are different: neither the conspicuous cylindrical sclerites and spindles forming the external layer of the coenenchyme (Fig. 3A, B) nor the internal coenenchyme radiates (Fig. 3C) of *Rhodolitica* are present in *Rhodelinda*, and the anthocodial points are shorter and sharper in *Rhodolitica* (Fig. 3D) (Pastorino and Ituarte 1996). As in *Rhodolitica*, *Stragulum* does not have inseparably fused sclerites forming the anthosteles. The types of sclerites found in the basal layer of the coenenchyme in *Stragulum* are similar to those that form the stolons in *Rhodolitica*; however, *Stragulum* colonies form encrusting sheets instead of stolons. Also, the characteristic anthostelar cylindrical sclerites of *Rhodolitica* are not present. Phylogenetically, *Tubipora* Linnaeus, 1758, in the family Tubiporidae, is also close to the above mentioned genera, but morphologically it is different from the other three genera. The anthostele walls of *Tubipora* form rigid tubes of solidly fused sclerites and the polyp tubes are joined laterally by an elevated series of transverse platforms (Fabricius and Alderslade 2001), producing complex structures. The genus is also zooxanthellate (Fabricius and Alderslade 2001) in contrast to the other three.

The morphological distinction of *Rhodolitica* from *Rhodelinda*, *Stragulum*, and *Tubipora* is also supported by phylogenetic analyses. Although all analyses strongly supported the placement of *Rhodolitica* in a clade with *Rhodelinda*, *Paratelesto*, *Stragulum*, and *Tubipora*, the relationships among those genera remained poorly resolved. Lack of clear evidence for a sister relationship between *Rhodolitica* and any one of these other related but morphologically distinct genera supports the decision to establish a new genus for *R. occulta* sp. nov., and highlights the importance of the integrative approach to octocoral taxonomic work.

## Supplementary Material

XML Treatment for Rhodolitica

XML Treatment for Rhodolitica occulta

## Supplementary materials

Supplementary material 1 Table S1. Sequence data used in phylogenetic analysis Data type Phylogenetic Explanation note **Table S1** contains the sequence data of Octocorallia used in the phylogenetic analysis for a new genus and species: *Rhodolitica occulta* gen. nov. sp. nov. https://binary.pensoft.net/file/532553This dataset is made available under the Open Database License (http://opendatacommons.org/licenses/odbl/1.0/). The Open Database License (ODbL) is a license agreement intended to allow users to freely share, modify, and use this Dataset while maintaining this same freedom for others, provided that the original source and author(s) are credited.Odalisca Breedy, Leen van Ofwegen, Catherine S. McFadden, Catalina Murillo-Cruz

Supplementary material 2 Fig. S1 Data type Phylogenetic Explanation note Maximum likelihood tree for mtMutS (738 nt). Only the stoloniferan clade that includes *Rhodolitica occulta* gen. nov. sp. nov. is shown, rooted to *Clavularia inflata*. Numbers on branches are bootstrap values (% of 100 replicates) https://binary.pensoft.net/file/532554This dataset is made available under the Open Database License (http://opendatacommons.org/licenses/odbl/1.0/). The Open Database License (ODbL) is a license agreement intended to allow users to freely share, modify, and use this Dataset while maintaining this same freedom for others, provided that the original source and author(s) are credited.Odalisca Breedy, Leen van Ofwegen, Catherine S. McFadden, Catalina Murillo-Cruz

Supplementary material 3 Fig. S2 Data type Phylogenetic Explanation note Maximum likelihood tree for COI (693 nt). Only the stoloniferan clade that includes *Rhodolitica occulta* gen. nov. sp. nov. is shown, rooted to *Clavularia inflata*. Numbers on branches are bootstrap values (% of 100 replicates). https://binary.pensoft.net/file/532555This dataset is made available under the Open Database License (http://opendatacommons.org/licenses/odbl/1.0/). The Open Database License (ODbL) is a license agreement intended to allow users to freely share, modify, and use this Dataset while maintaining this same freedom for others, provided that the original source and author(s) are credited.Odalisca Breedy, Leen van Ofwegen, Catherine S. McFadden, Catalina Murillo-Cruz

Supplementary material 4 Fig. S3 Data type Phylogenetic Explanation note Maximum likelihood tree for 28S rDNA (813 nt). Only the stoloniferan clade that includes *Rhodolitica occulta* gen. nov. sp. nov. is shown, rooted to *Clavularia inflata*. Numbers on branches are bootstrap values (% of 100 replicates). https://binary.pensoft.net/file/532556This dataset is made available under the Open Database License (http://opendatacommons.org/licenses/odbl/1.0/). The Open Database License (ODbL) is a license agreement intended to allow users to freely share, modify, and use this Dataset while maintaining this same freedom for others, provided that the original source and author(s) are credited.Odalisca Breedy, Leen van Ofwegen, Catherine S. McFadden, Catalina Murillo-Cruz
