# Supplementary material for: Rhodolitica on rhodoliths: a new stoloniferan genus (Anthozoa, Octocorallia, Alcyonacea)
Source: Zookeys. 2021 Apr 16;1032:63–77. doi: 10.3897/zookeys.1032.63431 (PMC8065023; doi:10.3897/zookeys.1032.63431)
Supplement: Supplementary material 2 — Fig. S1 [file zookeys-1032-063-s002.pdf]

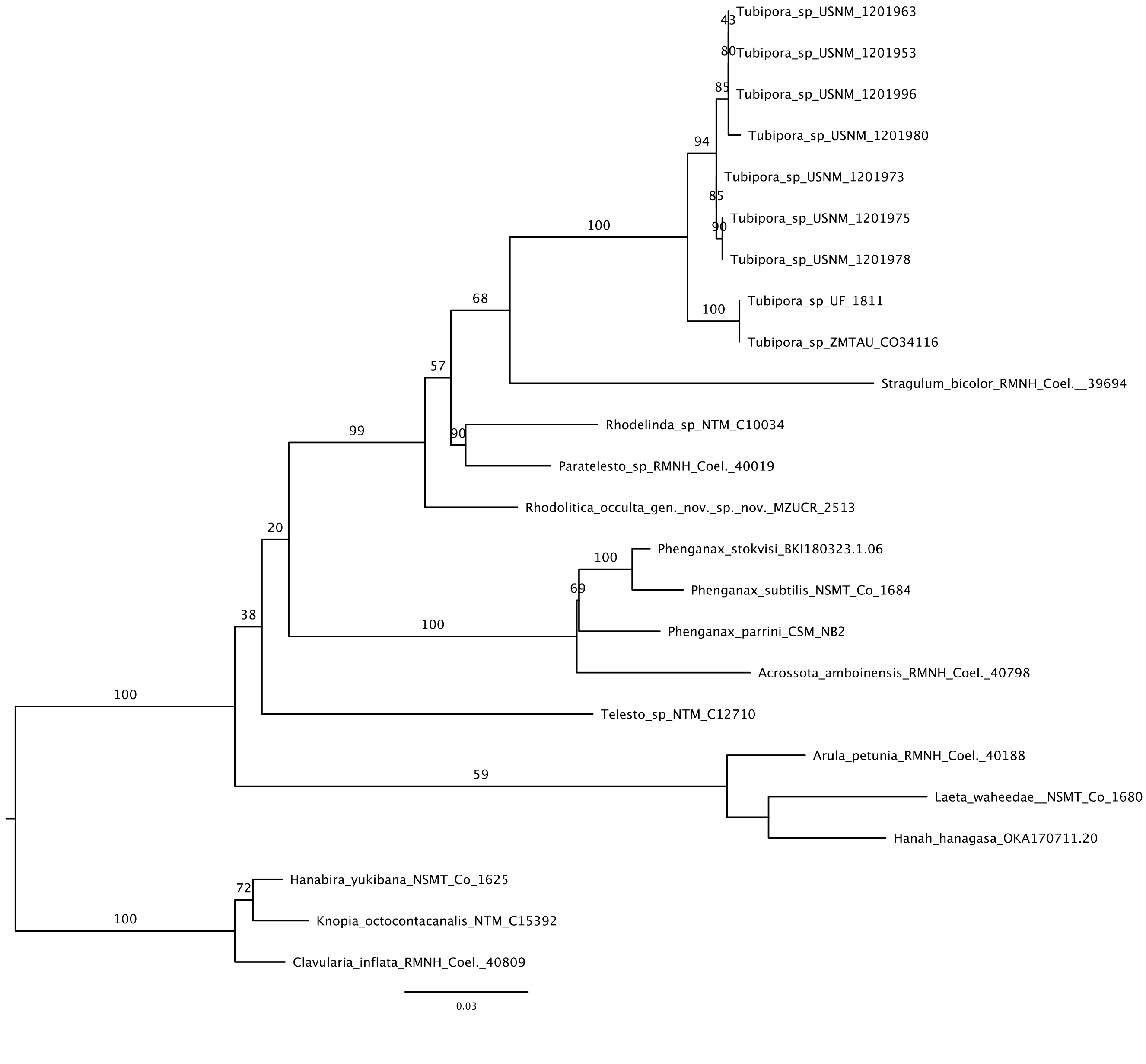

Supplemental Figure S1: Maximum likelihood tree for *mtMutS* (738 nt). Only the stoloniferan clade that includes *Rhodolitica occulta* gen. nov. sp. nov. is shown, rooted to *Clavularia inflata*. Numbers on branches are bootstrap values (% of 100 replicates).
